# Evaluation of the antibacterial and antibiofilm effect of mycosynthesized silver and selenium nanoparticles and their synergistic effect with antibiotics on nosocomial bacteria

**DOI:** 10.1186/s12934-024-02604-w

**Published:** 2025-01-04

**Authors:** Nahed Fathallah Fahmy, Marwa Mahmoud Abdel-Kareem, Heba A. Ahmed, Mena Zarif Helmy, Ekram Abdel-Rahman Mahmoud

**Affiliations:** 1 Microbiology and Immunology Department, Faculty of Medicine, Sohag University, Sohag, Egypt; 2 Botany and Microbiology Department, Faculty of Science, Sohag University, Sohag, 82524 Egypt; 3 Clinical and Chemical Pathology Department, Faculty of Medicine, Sohag University, Sohag, Egypt; 4 General Laparoscopic and Oncology Surgical Department, Sohag University Hospital, Sohag, Egypt

**Keywords:** Silver and selenium nanoparticles, *Aspergillus carneus*, Characterization, Antibacterial, Synergistic effect, Antibiofilm

## Abstract

**Background:**

The healthcare sector faces a growing threat from the rise of highly resistant microorganisms, particularly Methicillin-resistant *Staphylococcus aureus* (MRSA) and multidrug-resistant *Pseudomonas aeruginosa* (MDR *P. aeruginosa*). Facing the challenge of antibiotic resistance, nanoparticles have surfaced as promising substitutes for antimicrobial therapy. Recent studies showcase the effectiveness of various fungi species in nanoparticle synthesis. Mycosynthesized silver nanoparticles (AgNPs) and selenium nanoparticles (SeNPs) using *Aspergillus carneus* MAK 259 has been investigated and demonstrate antibacterial, antibiofilm and synergistic activities against (MRSA) and (MDR *P. aeruginosa*).

**Results:**

In the current research, silver nanoparticles (AgNPs) and selenium nanoparticles (SeNPs) were produced extracellularly using *A. carneus* MAK 259 culture supernatants. Colour change, an initial evaluation of the production of AgNPs and SeNPs. Then, UV absorption peaks at 410 nm and 260 nm confirmed the production of AgNPs and SeNPs, respectively. AgNPs and SeNPs were dispersed consistently between 5‒26 nm and 20–77 nm in size, respectively using TEM. FT-IR analysis was used for assessing proteins bound to the produced nanoparticles. The crystallinity and stability of AgNPs and SeNPs was confirmed using X-ray diffraction analysis and zeta potential measurements, respectively. Antibacterial, antibiofilm and synergistic effects of both (NPs) with antibiotics against MRSA and MDR *P. aeruginosa* were tested by Agar well diffusion, tissue culture plate and disc diffusion method respectively. Both (NPs) inhibited the growth of *P. aeruginosa* more than *S. aureus*. But, SeNPs was stronger. AgNPs had stronger antibiofilm effect especially on biofilms producing *S. aureus*. as regard synergestic effects, Both (NPs) had higher synergestic effects in combination with cell wall inhibiting antibiotics against *P. aeuroginosa* While, on *S. aureus* with antibiotics that inhibit protein synthesis and affect metabolic pathways.

**Conclusions:**

Our study demonstrated that the mycosynthesized SeNPs had remarkable antibacterial effect while, mycosynthesized AgNPs exhibited a considerable antibiofilm effect. Both NPs exhibited higher synergistic effect with antibiotics with different modes of action. This approach could potentially enhance the efficacy of existing antibiotics, providing a new weapon against drug-resistant bacteria where the described silver and selenium nanoparticles play a pivotal role in revolutionizing healthcare practices, offering innovative solutions to combat antibiotic resistance, and contributing to the development of advanced medical technologies.

**Supplementary Information:**

The online version contains supplementary material available at 10.1186/s12934-024-02604-w.

## Background

Despite the discovery and development of multiple new antibiotics, The effectiveness of healthcare systems around the world is presently threatened by the highly resistance of MDR microorganisms. Methicillin resistant *Staphylococcus aureus* (MRSA) and multidrug resistance *Pseudomonas aeruginosa* (MDR *P. aeruginosa*) are two of the most well-known highly resistant bacteria [1, 2].

Methicillin-resistant *Staphylococcus aureus* (MRSA) is resistant to the beta-lactam class of antibiotics. Methicillin is one of these antibiotics, along with others that are more widely used, like oxacillin, penicillin, and amoxicillin [3], While multidrug resistance *Pseudomonas* was defined as Resistance to at least three antibiotics from various classes, including antipseudomonal penicillins, aminoglycosides, cephalosporins, fluoroquinolones and carbapenems [4].

Nanoparticles have been suggested as a therapy for infections due to the need for novel antimicrobial medications. Since They are substantially less harmful to human cells and kill bacteria in various ways than traditional antibiotics. Therefore, nanomaterials may be considered as a potential substitute for antibiotics in the treatment of bacterial illnesses [5, 6].

Silver and selenium nanoparticles possess many distinct uses. Owing to their characteristics, silver nanoparticles (AgNPs) possess applications in the medical and catalysis fields [7]. The antioxidant, anticancer, catalytic, and antibacterial activity of silver nanoparticles has been thoroughly demonstrated. AgNPs are particularly renowned for their superior conductivity and antimicrobial effects, which have been used in medical settings such as surgical implants, wound healing, and dental prosthetics [8]. In addition Several researches in recent years have demonstrated the potential of selenium nanoparticles (SeNPs) to display anticancer, antioxidant, antibacterial, and anti-biofilm capabilities [9].

Although there are numerous physical and chemical methods for creating nanomaterials, they usually necessitate high temperatures, toxic chemicals, and the release of extremely unsafe by-products [10]. Researchers have recently become interested in the use of biomaterials in order to produce nanoparticles.

Using microorganisms in the manufacture of nanomaterials is regarded as the ideal source. Discovering new microbial strains and evaluating their capacity for nanomaterial synthesis has become a rapidly expanding and exciting research field for considering potential future advancements in the process of nano-production [11].

Fungi compared to other microbes may create significantly more metabolites, making them better suited for nanopartices synthesis [12]. The ability of several fungi to produce extremely large rates of secreted proteins assists in accelerating up the creation of nanoparticles, due to the reality that they have mycelia, with larger surface areas, promoting metal ion-fungal reducing agent interactions, as highlighted by El-Ramady et al. [13]. In recent times, extracts from various fungi species have been utilized for the production of metallic nanoparticles [14–19].

Till date, no report has present about mycological synthesis of AgNPs and SeNPs using *A. carneus* MAK 259. Thus, the objective of this study was to synthesize and characterize these nanoparticles, as well as evaluate their antibacterial and antibiofilm effects, in order to determine which has a stronger effect against MRSA and MDR *P. aeruginosa* isolates from Sohag University Hospital. Furthermore, the study aimed to investigate the potential synergistic effect of combining these nanoparticles with antibiotics.

## Materials and methods

### Chemicals and microorganism source

The tested *Aspergillus* strain was isolated from cultivated soil samples on glucose- Czapek Dox agar medium and kept on PDA slants at 4 °C For long-time storage. 46 multidrug resistant *Pseudomonas aeruginosa* (MDR *P. aeruginosa*) and 36 methicillin resistant *Staphylococcus aureus* (MRSA) isolates which were recovered from surgical departments, Sohag University Hospital from June 2022 to April 2023. Silver nitrate (AgNO_3_) and sodium selenite (Na_2_SeO_3_) were purchased from Sigma-Aldrich Chemicals.

### Genotypic identification of the tested fungal strain

#### DNA extraction

To extract DAN (DNA Analogous Nucleotides), the fungus strain *Aspergillus* MAK 259 was inoculated on potato dextrose broth. The mixture was then placed in a shaker incubator at 200 rpm and kept at 28 °C for 5 days. During this time, the fungus formed pellets. The process of obtaining the complete genomic DNA followed the methodology mentioned by Rasmey et al. [20].

#### PCR amplification

Employing the two primers 18SF: 5/-TTAAGCCATGCATGTCTAAG-3/ (forward) and 18SR: 5/-GACTACGACGGTATCTAATC-3/ (reverse), the gene encoding for 18S rRNA was amplified through the polymerase chain reaction (PCR) from isolated genomic DNA. The PCR amplification was conducted using the Qiagen Proof-Start Tag Polymerase Kit (Qiagen, Hilden, Germany), with a substrate mixture consisting of approximately 50 ng of DNA template, 12.5 µL of PCR Master Mix, 5 pmol (0.5 µL) of both forward and reverse primers, and 11.5 µL of DNAase-free water for a total volume of 25 µL. This action was carried out on the ice. The entire reaction mixture was subsequently incubated in a controlled thermal cycle (Master cycler, Eppindorff, Germany), which involved 35 cycles of denaturation at 94 °C for 30 s, followed by annealing at 52 °C for 30 s, and extension at 72 °C for 3 min, in order to accomplish the synthesis of all strands. The reaction was then halted by cooling at 4 °C. Gel electrophoresis on 1% (v/v) agarose TBE-gels (Tris-base Boric EDTA-gels) was performed to examine the PCR products. The gels were documented and viewed under UV light. Purification of PCR products of approximately 1100 bp was carried out from the gel using the QIA quick gel extraction kit from Qiagen, Hilden, Germany.

#### DNA sequencing

To sequence the generated PCR fragments, an automated DNA sequencer (3500 Genetic Analyzer from Applied Biosystems, Thermo Fisher) was applied in order to determine the similarity between obtained sequences and previously published ones. This is accomplished by comparing the full-length sequences using BLAST on the NCBI website: http://www.ncbi.nlm.nih.gov/BLAST/. To further analyze the data, CLUSTALX (http://clustalw.ddbj.nig.ac.jp/top-ehtml) was utilized to compare multiple sequences, and then MEGA 7.2.2 was used to construct a Maximum Parsimony (MP) plot. The resulting data, including the 18S rRNA gene sequence from isolate MAK 259 and other sequences from the Gen-Bank database [21], was then used to create a phylogenetic tree using the software MEGA 7. For analyzing the evolutionary history, the neighbour-joining approach proposed by Saitou and Nei [22], along with the maximum composite likelihood method, introduced by Tamura et al. [23] to calculate the evolutionary distances.

### Mycosynthesis of silver and selenium nanoparticles

To cultivate and facilitate the growth of fungi to generate silver and selenium nanoparticles, a modified version of the methods outlined by Xue et al. [24] was employed. Specifically, *A. carneus* MAK 259 was cultivated in Potato Dextrose Broth (PDB) under aerobic conditions at a temperature of 28 ± 2 °C for a duration of 10 days. The resulting biomass was collected by filtering it through Whatman Filter Paper No. 1 and subsequently washed with distilled water to eliminate any residual components from the growth medium. Subsequently, 10 g of the moist biomass was placed in flasks containing 100 mL of water. Following an incubation period of 48 h at a temperature of 28 °C and with orbital shaking at 120 rpm, the sample was filtered using Whatman filter paper No. 1. The resulting filtrate was then utilized to generate silver and selenium nanoparticles in the following manner.

Silver nitrate (AgNO_3_) and sodium selenite (Na_2_SeO_3_) (1 mM) were individually instilled into the filtrate to facilitate the formation of AgNPs and SeNPs, respectively. The proportion of cell filtrate to AgNO_3_ and Na_2_SeO_3_was maintained at 1:9 (v/v), and the reaction mixtures were incubated at 28 °C for 24 h. Controls (without addition of AgNO_3_ or Na_2_SeO_3_) were used.

### Characterization of biogenic nanoparticles

Initial observation of a color transformation into brown and red orange is the first indication for the production of AgNPs and SeNPs individually in the reaction medium. The reduction of silver and selenium ions was affirmed by UV–vis spectrophotometer (JENWAY 7315 spectrophotometer, Staffordshire, UK.), at wavelengths of 300–700 nm in case of AgNPs and 200–800 nm in case of SeNPs. The structure and particle size of both nanoparticles were investigated using a TEM examination technique (TEM, Electron Microscope Unit, Assiut University, Egypt), images were captured randomly. Further investigation into the biomolecules responsible for reducing, capping, and stabilizing AgNPs and SeNPs was conducted through Fourier transform infrared spectroscopy(ALPHA II, with platinum ATR, Germany) with readings taken in the range of 4000–400/cm using the pure potassium bromide pellet technique. Additionally, the crystallinity of AgNPs and SeNPs was assessed through X-ray diffraction (XRD) analysis, with a diffraction pattern evaluated at 2θ = 28 (30–80°) at 40 keV energy level using a D8 Advanced Bruker model. The diffractometer operates at 1.5406 Å wavelength, 40 kV, and 40 mA generator. Both biosynthesized nanoparticles stability was measured by determination of zeta potential using particle size analyzer (Zetasizer Nano ZN, Malvern Panalytical Ltd, United Kingdom) at fixed angle of 173° at 25 °C.

### Sample collection and identification of bacterial isolates

#### Sampling

This is a cross-sectional study performed in Medical Microbiology and Immunology Department, Faculty of Medicine, Central research laboratory, Surgery department, Sohag University hospital in the period from June 2022 to April 2023. The study included *S. aureus* and *P. aeruginosa* isolates only that are identified as MRSA and MDR-*P. aeruginosa*, respectively. Pus samples were collected from patients admitted to surgical departments including orthopaedic, vascular, plastic, and general surgery, urology and oncology who develop surgical site infections. Pus collected by using sterile cotton swabs. Every patient or their family member gave their informed consent. The Sohag University Faculty of Medicine's ethical committee granted ethical approval.

#### Identification of *Staphylococcus aureus*

Pus samples were quickly transferred and immediately inoculated on nutrient agar, blood agar and mannitol salt agar (CONDA, Spain) plates. The plates were incubated for 24–48 h at 37 °C. *S. aureus* produced golden yellow colonies on nutrient agar; produced β-hemolysis on blood agar, and produced yellow colonies on mannitol salt agar. Gram stained smears were examined Microscopically, *S. aureus* was identified as Gram positive cocci arranged in grape-like clusters. Biochemical reactions were performed on the isolates, and *S. aureus* proved positive for catalase, coagulase, and DNase.

#### Identification of ***Pseudomonas aeruginosa***

Pus samples were transported rapidly and inoculated on cetrimide agar (CONDA, Spain) then aerobically incubated at 37 °C for 24–48 h. Visual examination revealed The pigmentation ranged from yellow-green to blue, indicating the synthesis of pyocyanin. Colonies were examined Under ultraviolet (UV) light to detect fluorescein. *P. aeruginosa* strains generally generate both pyocyanin and fluorescein. Gram stained smears showed gram negative bacilli, biochemically isolates were Oxidase positive. Identification was confirmed by API 20 NE (bioMérieux, France).

### Antibiotic sensitivity test

#### Antibiotic sensitivity test of *S. aureus*

According to CLSI guidelines (CLSI, 2022) [25], AST was performed by modified Kirby-Bauer method on Mueller–Hinton agar (Himedia, India) using the following antibiotic discs: gentamicin 10 μg, erythromycin 15 μg, tetracycline 30 μg, ciprofloxacin 5 μg, nitrofurantoin 300 μg, clindamycin 2 μg, trimethoprim-sulfamethoxazole 1.25/23.75 μg, chloramphenicol 30 μg, rifampin 5 μg, quinapristin-dalphopristin 15 μg, linezolid 30 μg, cefoxitin 30 μg. Inhibition zone diameters were measured in millimetres (mm), and the results were interpreted as resistant (R), intermediate (I), and sensitive (S). *Staph aureus* that is resistant to cefoxitin (R) is known as methicillin-resistant *S. aureus* [26].

#### Antibiotic sensitivity test (AST) of *P. aeruginosa*

According to CLSI guidelines **(CLSI, 2022),** On Mueller–Hinton agar, AST was done using a modified Kirby-Bauer technique by using the following antibiotic discs: pipercillin 100 μg, pipercillin + tazobactam 100/10 μg, ceftazidime 30 μg, aztreonam 30 μg, impenem 10 μg, meropenem 10 μg, gentamicin 10 μg, ciprofloxacin 5 μg. colistin. Inhibition zone diameters were measured in millimetres (mm) and classified as sensitive (S), intermediate (I), and resistant (R). MDR-*P. aeruginosa* is defined as *P. aeruginosa* that is Intermediate (I) or Resistant (R) to at least one medication in at least three of the following five categories: (1) extended spectrum penicillin (pipercillin, pipercillin + tazobactam), (2) extended-spectrum cephalosporin, (3) carbapenems, (4) fluoroquinolones, (5) aminoglycosides [26].

### The tissue culture plate technique for detecting biofilm development

*P. aeuriginosa* and *S. aureus* isolates were examined for biofilm development by tissue culture plate technique. Brain heart infusion "BHI" broth (Himedia, India) supplemented with 1% glucose was used to develop the isolates and incubated for 18 h at 37 °C. The culture was then diluted with non-inoculated BHI broth (1 in 100) then Each well of the 96 flat-bottomed, sterile polystyrene microtitre plate was injected with 200 μL of the diluted bacterial suspensions. After 24 h of incubation at 37 °C, the contents of the wells were carefully removed and rinsed three times with 200 μL of phosphate buffered saline (pH 7.2), then dried. After adding 200 μL of 0.1% crystal violet stain to each well, the plates were allowed to sit at room temperature for 10 min. The wells of the plate were rinsed with deionized water 3 times and finally solubilized in ethanol 95%. The absorbance was measured at 600 nm using ELISA reader. Negative control was used by adding 200 μL of sterile non inoculated broth. The assay is performed in triplicate. Biofilm formation was classified based upon the mean optical density (OD) of each bacterial film as follow; non biofilm (<0.062), weak (0.062–<0.124), moderate (0.124–0.248) and strong (>0.248) [27]. The cut-off optical density (OD) for a tissue culture plate is three standard deviations (SD) above the negative control's mean OD.

### Antibacterial effect of AgNPs and SeNPs

Using the agar well diffusion technique, the antibacterial efficacy of AgNPs and SeNPs against isolates of *S. aureus* and *P. aeruginosa* was investigated. 0.5 McFarland bacterial suspensions were prepared from the tested isolates and inoculated on the surface of MHA plates using sterilized cotton swabs. Using a cork borer, 3 mm diameter wells were created on Mueller–Hinton agar (17.5 g peptone, 17.0 g agar, 3.0 g beef infusion, 1.5 g starch, pH 7.4 ± 0.2). Two-hundred μL of AgNPs (250 μg/mL) and SeNPs (250 μg/mL) were placed into each well of the MHA plates independently. After 24 h of incubation at 37 °C, the plates were examined to see if a distinct inhibitory zone had formed. These tests were performed in triplicate [14].

### Synergstic effect of AgNPs and SeNPs with antibiotics

To study the synergistic effect of AgNPs and SeNPs when used with antibiotics, we used the standard antibiotic discs recommended by CLSI, 2022 for *S. aurues* and *P. aeurginosa* as previously listed in AST. The disk diffusion method was performed on Müller-Hinton agar inoculated with 0.5 McFarland standard of bacterial suspension of the tested isolates, by using two types of discs; (1) antibiotic discs saturated with 10 μL of AgNPs, (2) antibiotic discs saturated with 10 μL of SeNPs. The plates were incubated at 35 °C for 24 h, following which the inhibition zones were measured in millimetres and compared to the CLSI, 2022 standard antibiotics inhibition zones. This experiment was carried out in triplicate. This method was used to estimate the fold increase in the diameter of the inhibition zone of each antibiotic following combination with AgNPs and SeNPs according to this formula;

The fold increase = (b − a)/a × 100, Where; (a) is the inhibition zone of antibiotic alone and (b) is the inhibition zone of antibiotic plus nanoparticles (AgNPs or SeNPs). This formula was used to assess how much the antibiotic, in combination with AgNPs or SeNPs, increases the inhibition zone surrounding the tested organism [28].

### Biofilm inhibition by AgNPS and SeNPs

Biofilm inhibition by nanoparticles on *P. aeuriginosa* and *S. aureus* isolates was carried out as previously described by tissue culture plate method in 96 well polystyrene microtitre plates using different concentration of nanoparticles (250, 125, 62.5, 31.2, 15.6, 7.8, 3.9, 1.9 and 0.9 μg/mL). By serial two-fold dilutions, different concentrations of AgNPs and SeNPs were generated from a stock concentration of 250 g/mL, with the lowest concentration applied was 0.9 g/mL To identify the minimum inhibitory concentration (MIC) of AgNPs and SeNPs against biofilm formation. Bacterial suspensions were prepared by adding 10 μL of the test pathogens to 180 μL of BHI" broth in individual wells of the plates. Different concentrations of nanoparticles prepared by the two fold serial dilution were tested by adding 10 μL of AgNPs and 10 μL of SeNPs to the bacterial suspensions in separate wells and thoroughly mixed, the plates were incubated for 24 h at 37 °C. Then, The contents of the microtiter plate wells had been eliminated and rinsed with PBS, the plates dried in air for 45 min. 2% w/v sodium acetate used for fixation of adherent bacteria in the wells. The wells were stained with 200 μL of crystal violet dye and incubated in the dark for half an hour. Deionized water was used to wash the wells until any extra colour was eliminated. After this, we added 200 μL of 95% ethanol to each well and then absorbance was calculated at 600 nm. A negative control was used, which was sterile broth. As untreated controls, bacterial suspensions devoid of AgNPs or SeNPs were utilized. Experiments were carried out in triplicate [29].

The percentage of biofilm inhibition is calculated using the following equation [30]:

$$\left[ {{1} - \left( {{\text{A6}}00\,{\text{of}}\,{\text{cells}}\,{\text{treated}}\,{\text{with}}\,{\text{AgNPs}}\,{\text{or}}\,{\text{SeNPs}}/{\text{A6}}00\,{\text{of}}\,{\text{non}} - {\text{treated}}\,{\text{control}}\,{\text{cells}}} \right)} \right] \times {1}00.$$

### Analytical statistics

Data was analyzed using STATA version 17.0 (Stata Statistical Software: Release 17.0 College Station, TX: StataCorp LP.). The mean, standard deviation, or median and range were used to express quantitative data. To compare the means of two groups, the data was analysed using the student t-test. In order to compare several measurements of the same sample, the Wilcoxon signed-rank test was applied. The frequency and percentage were used to display the categorical data. *p* value < 0.05 was considered statistically significant.

## Results

### Genotypic identification of the fungal strain

**Fig. 1:**
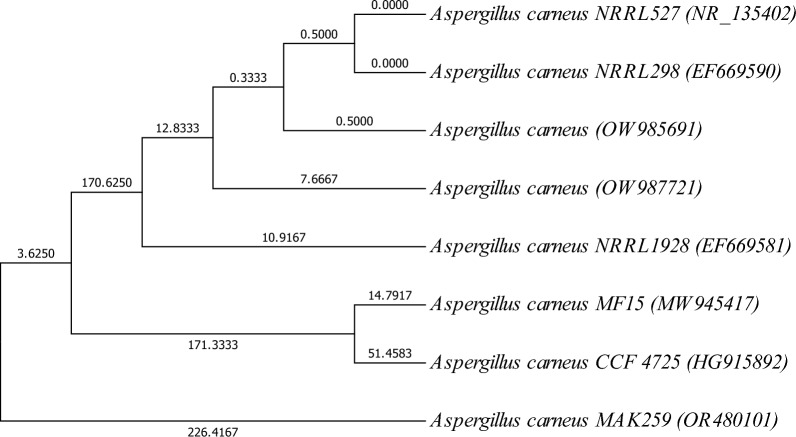
Evolutionary relationships of *Aspergillus carneus* MAK 259 (OR480101) with the other related *Aspergillus* strains in GenBank database

The genotypic identification of the *Aspergillus* MAK 259 strain was performed by analyzing the nucleotide sequence of the amplified 18S rRNA gene. The results indicate that this isolate shares 99% similarity with *Aspergillus carneus* strain CCF 4725 (HG915892), indicating their identical nature. Figure 1 illustrates the position of this isolate among the closely related *Aspergillus* species using the neighbour-joining method. The evolutionary history was inferred using the Maximum Parsimony method, resulting in the most parsimonious trees with a length of 671. The consistency index, retention index, and composite index were calculated as 0.964232 (0.951417), 0.939241 (0.939241), and 0.905646 (0.893609), respectively, considering all sites and parsimony-informative sites (values in parentheses). The MP tree was obtained using the Subtree-Pruning-Regrafting (SPR) algorithm with a search level of 0, and initial trees were generated through the random addition of sequences (10 replicates). Branch lengths were determined using the average pathway method and are indicated alongside the branches, representing the number of changes across the entire sequence. The analysis involved 8 nucleotide sequences, including 1st + 2nd + 3rd + Noncoding codon positions. Positions containing gaps or missing data were excluded from the analysis, resulting in a final dataset of 531 positions. The evolutionary analyses were conducted using MEGA7 [21]. The nucleotide sequence of the 18S rRNA gene for *Aspergillus carneus* MAK 259 was submitted under the accession number OR480101 in the GenBank database.

### Mycological synthesis of AgNPs and SeNPs using *A. carneus* MAK 259

**Fig. 2:**
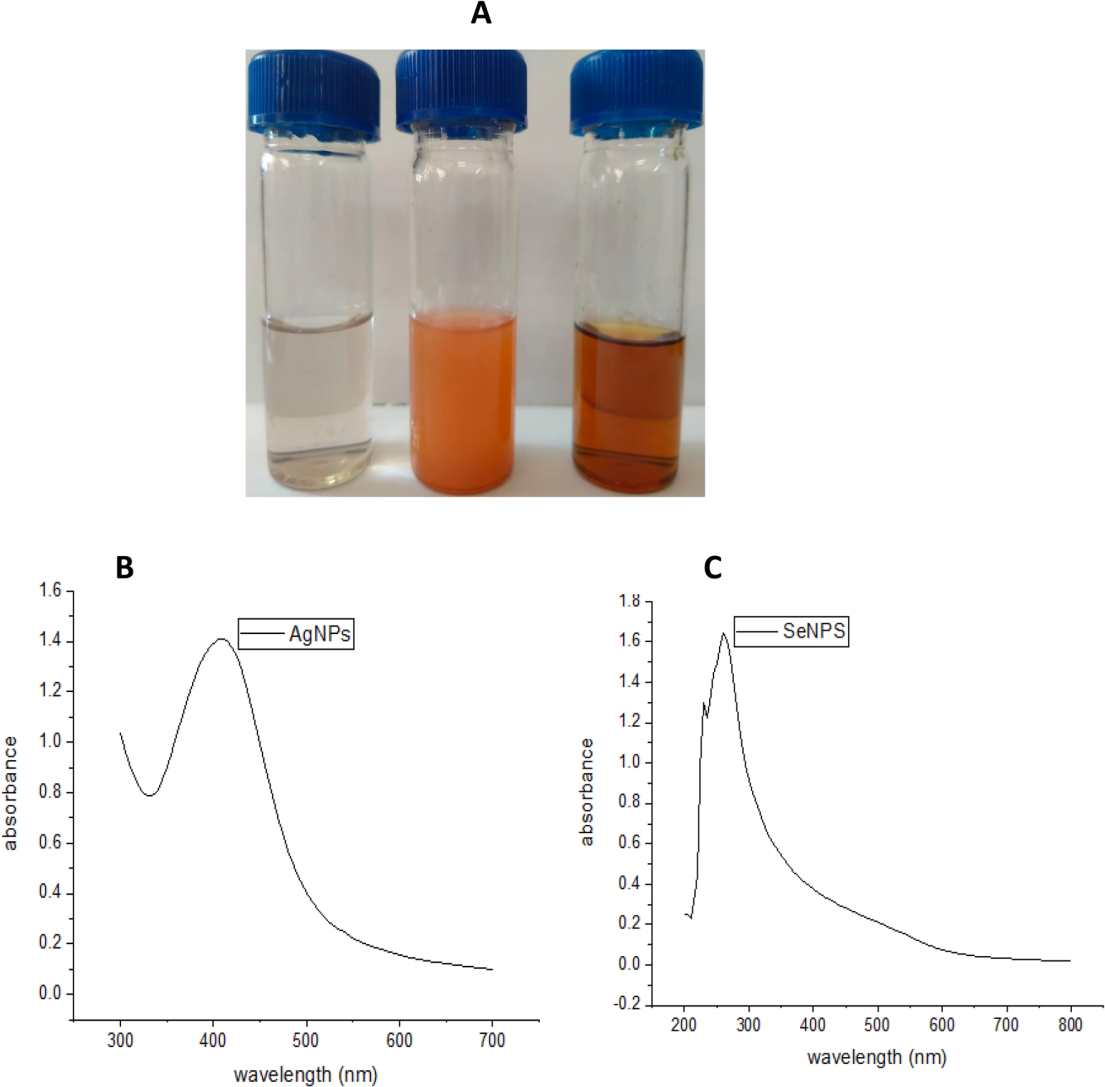
UV–Vis Spectroscopy pattern **A** Tubes showing the cell-free broth before and 24 h after treatment with 1 mM (Na_2_SeO_3_ and AgNO_3_) solutions (cell free broth with no color change, SeNPs tube with reddish orange color and AgNPs tube with brown color). **B** AgNPs UV– Vis Spectroscopy pattern. **C** SeNPs UV–Vis Spectroscopy pattern

The intent of this study was to create convenient one-pot methods for producing silver nanoparticles (AgNPs) and selenium nanoparticles (SeNPs). silver ions and selenite ions were separately exposed to culture filtrates of *Aspergillus carneus* MAK 259 (OR480101) (as depicted in Fig. 2A). Over a period of 24 h, the solutions gradually changed in color, turning brown for the AgNPs tubes and reddish orange for the SeNPs tubes. Conversely, the control tubes exhibited no alteration in color (Fig. 2A).

### Characterization of biogenic AgNPs and SeNPs

The analysis of silver nanoparticles (AgNPs) using UV–visible spectrophotometry indicate the presence of peak absorbance at 410 nm (as shown in Fig. 2B). On the other hand, for selenium colloidal suspensions, the maximum absorbance was observed at 260 nm (as depicted in Fig. 2C).

**Fig. 3:**
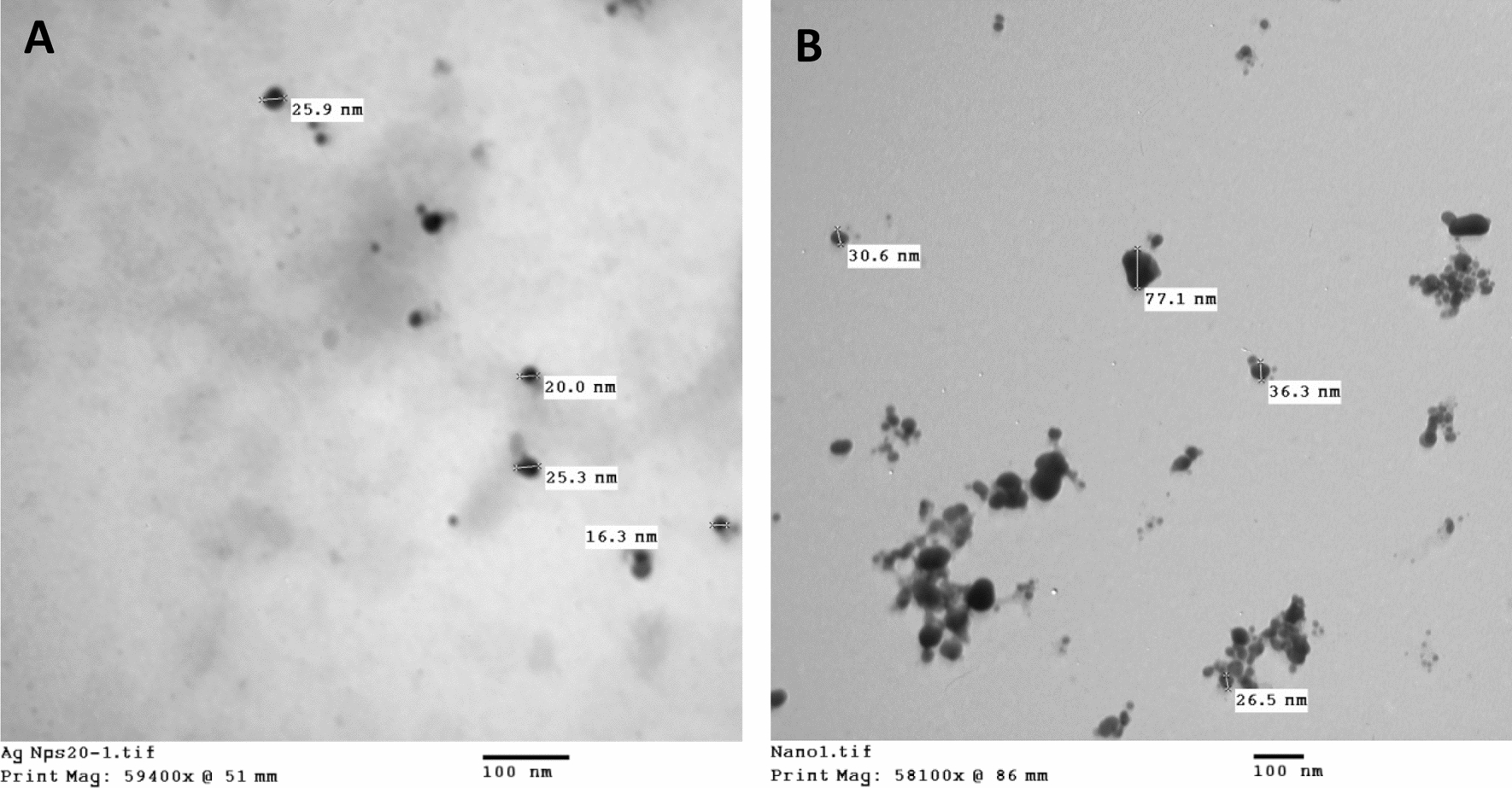
Transmission electron microscopic image of nanoparticles **A** AgNPs, **B** SeNPs (scale bar = 100 nm)

TEM images of AgNPs synthesized by *Aspergillus carneus* MAK 259 showed well-distributed, spherical and monodispersed AgNPs with size ranging between 5 and 26 nm (Fig. 3A). Also, TEM micrographs of SeNPs showed well dispersed, spherical particles of diameter ranged from 20 to 77 nm (Fig. 3B).

**Fig. 4:**
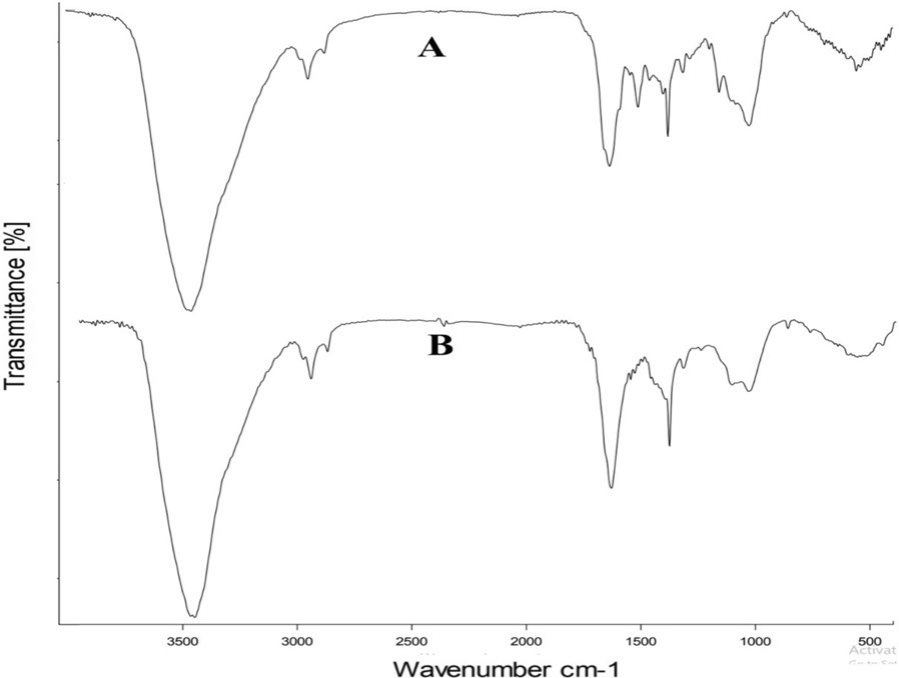
FT-IR analysis of biogenic nanoparticles **A** AgNPs, **B** SeNPs

The involvement of various functional groups in the synthesis and stabilization of the resulting nanoparticles (AgNPs and SeNPs) has been confirmed through Fourier-transform infrared (FT-IR) spectroscopy. This confirmation is evident from the spectra shown in Fig. 4A, B, which exhibit distinct absorption peaks for both AgNPs and SeNPs. Notably, a significant absorption peak was observed at 3424 cm^−1^, indicating the presence of specific functional groups. Additionally, the spectrum demonstrates the presence of a “C=C” stretch at approximately 1633 cm^−1^. Furthermore, sharp bands were detected at around 1404 cm^−1^ and 1384 cm^−1^. These observations indicate the presence of a C–N stretch, characterized by medium intensity peaks at ~ 1404 cm^−1^ and 1384 cm^−1^.

**Fig. 5:**
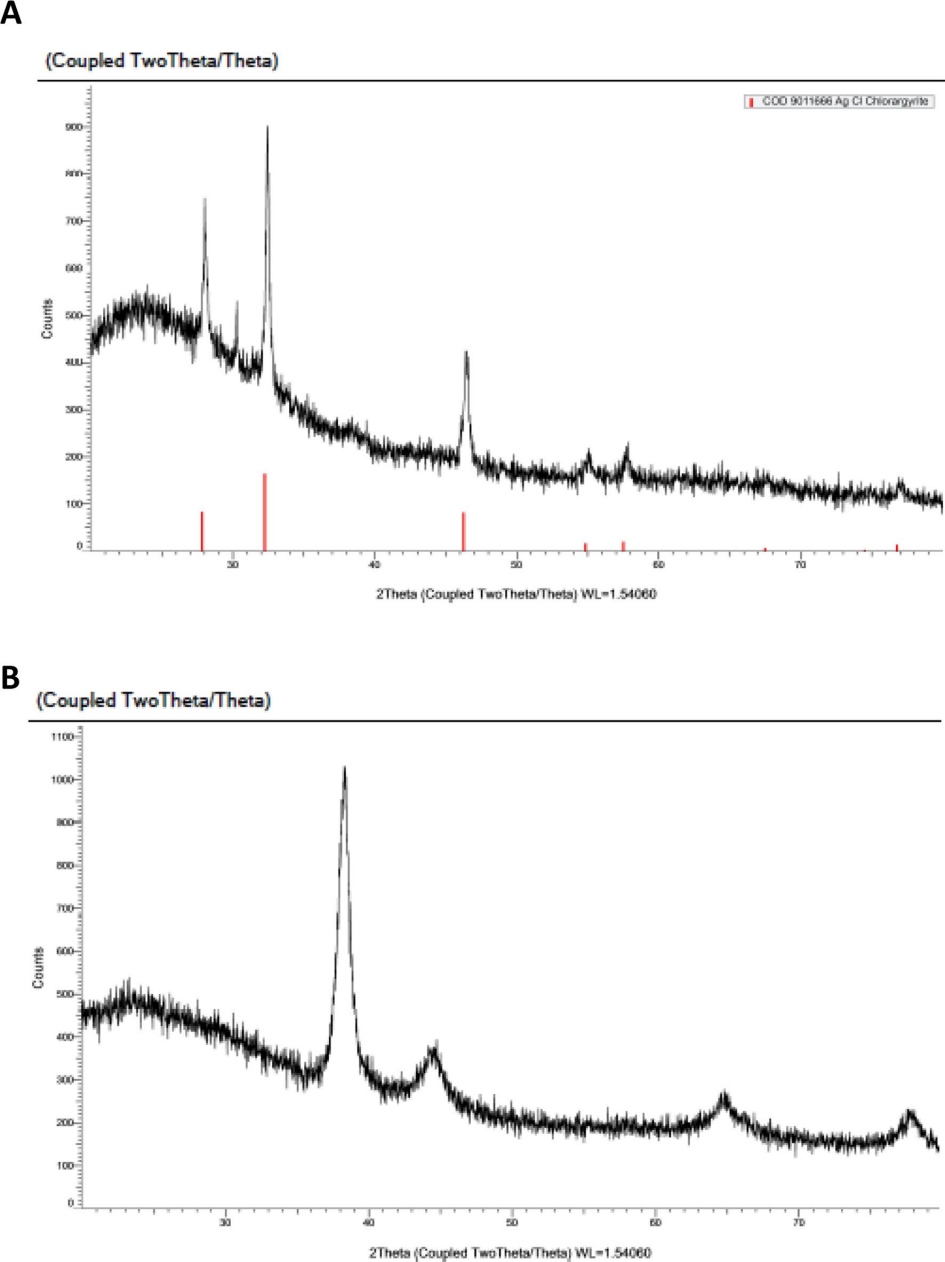
XRD pattern of mycosynthesized nanoparticles **A** AgNPs, **B** SeNPs

X-ray diffraction research at this point showed that the nature of synthesised AgNPs and SeNPs is crystalline (Fig. 5). The intensities were noted from 20° to 80°. The XRD pattern for AgNPs (Fig. 5A) indicated that the diffraction peaks that were visible at 2θ = 28°, 32°, 46°, 55°, 58° and 77° correspond to 100, 101, 111,112,200 and 311 and confirmed the crystalline phase of Ag.

Figure 5B displays the synthesised SeNPs' XRD pattern. According to the results of the XRD study, the planes of selenium have 111, 200, 220, and 311 as their respective 2θ values for 38, 44, 65, and 78 degrees.

**Fig. 6:**
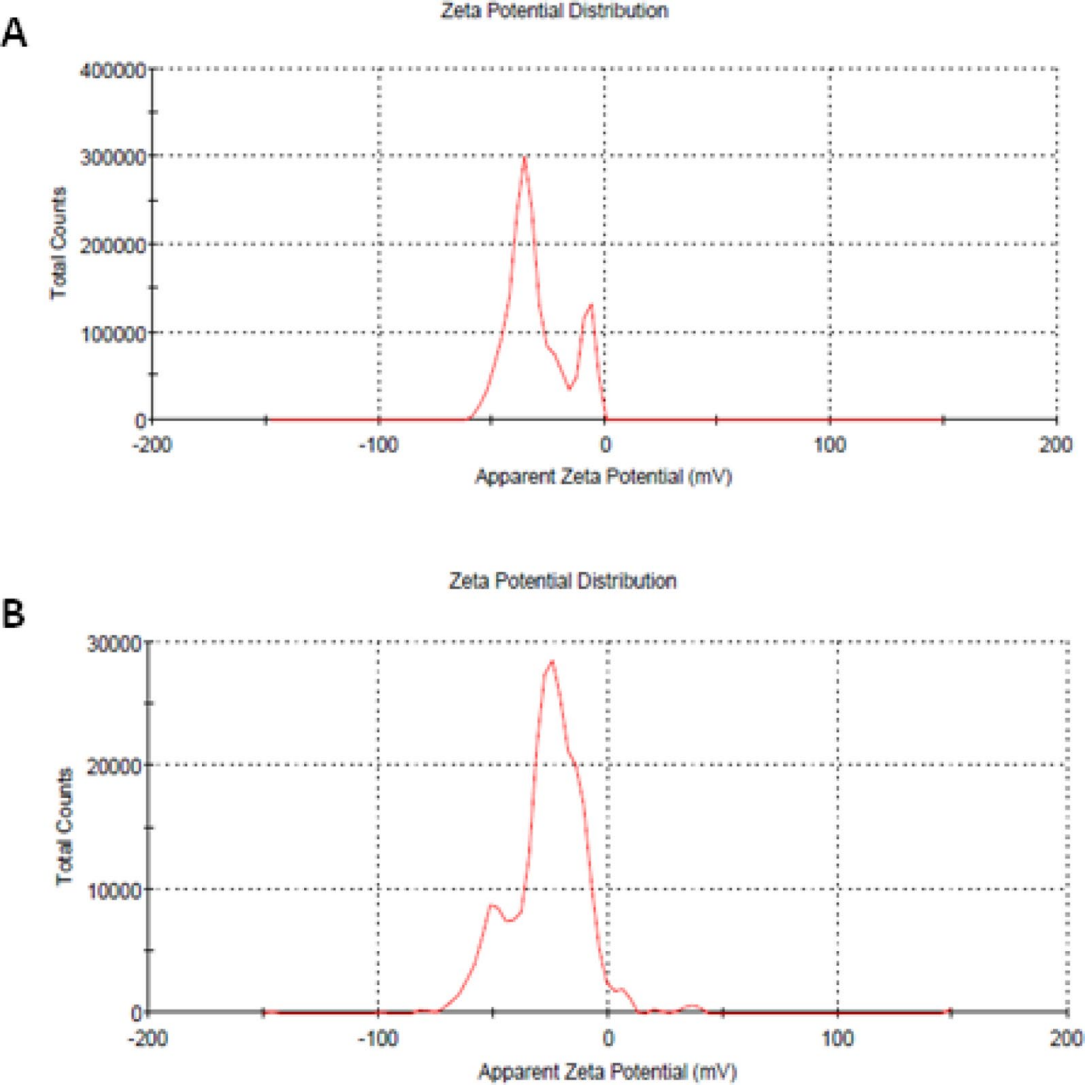
Zeta potential of **A** AgNPs, **B** SeNPs synthesized with *A. carneus* MAK 259

The stability of both AgNPs and SeNPs was also confirmed via zeta potential analysis as shown in Fig. 6A, B. Interestingly, AgNPs and SeNPs have negative potential of − 30 mV and − 25.7 mV, respectively.

### The antibacterial effect of nanoparticles

By comparing the ability of nanoparticles to inhibit both gram-positive and gram-negative bacteria, AgNPs had superior antibacterial efficacy against *P. aeruginosa* than *S. aureus *(*p value 0.0006*)*.* Also, SeNPs had the same results with a statistically significant difference (*p* value < 0.0001, Table 1). In this study, we aimed to detect which type of nanoparticles have a more powerful effect, we found that SeNPs had a more remarkable inhibiting impact on growth of *S. aureus* as well as *P. aeuroginosa* than AgNPS (*p* value < 0.0001, Table 1).

**Table 1:** The antibacterial effect of AgNPs and SeNPs on *S. aureus* and *P. aeuroginosa*

	*S. aureus* *N* = 36	*P. aeuroginosa* *N* = 46	*p* value
*The antibacterial effect of AgNPs*			
Mean ± SD	26.28 ± 2.98	29.83 ± 5.34	0.0006
Median (range)	26 (20:32)	30 (20:41)	
*The antibacterial effect of SeNPs*			
Mean ± SD	32.94 ± 3.01	37.52 ± 3.54	<0.0001
Median (range)	33 (27:38)	38 (28:45)	
*p* value compare AgNPs and SeNPs effect	<0.0001	<0.0001	

### Biofilm formation and the biofilm inhibition by nanoparticles

In the present study, 100% of *S. aureus* (36/36) were detected as a strong biofilm producers, while 91.3% of *P. aeruginosa* (42/46) were strong biofilm producers and 8.7% (4/46) were classified as moderate biofilm category.

The anti-biofilm effect of mycosynthesized AgNPs was tested in vitro on biofilm-producing *S. aureus* and *P. aeruginosa* in a dose-dependent manner, the assay's findings showed that certain AgNPs concentrations inhibited biofilm production by the tested organisms, in comparison to the experiment's negative control. The highest antibiofilm activity of AgNPs on *S. aureus* was detected at a concentration of 15.6 μg/mL, showing an approximate 94.36% inhibition percentage. However, on *P. aeruginosa*, higher concentration of AgNPs 62.5 μg/mL had the best biofilm inhibition capacity with inhibition value around 90.3%.

**Fig. 7:**
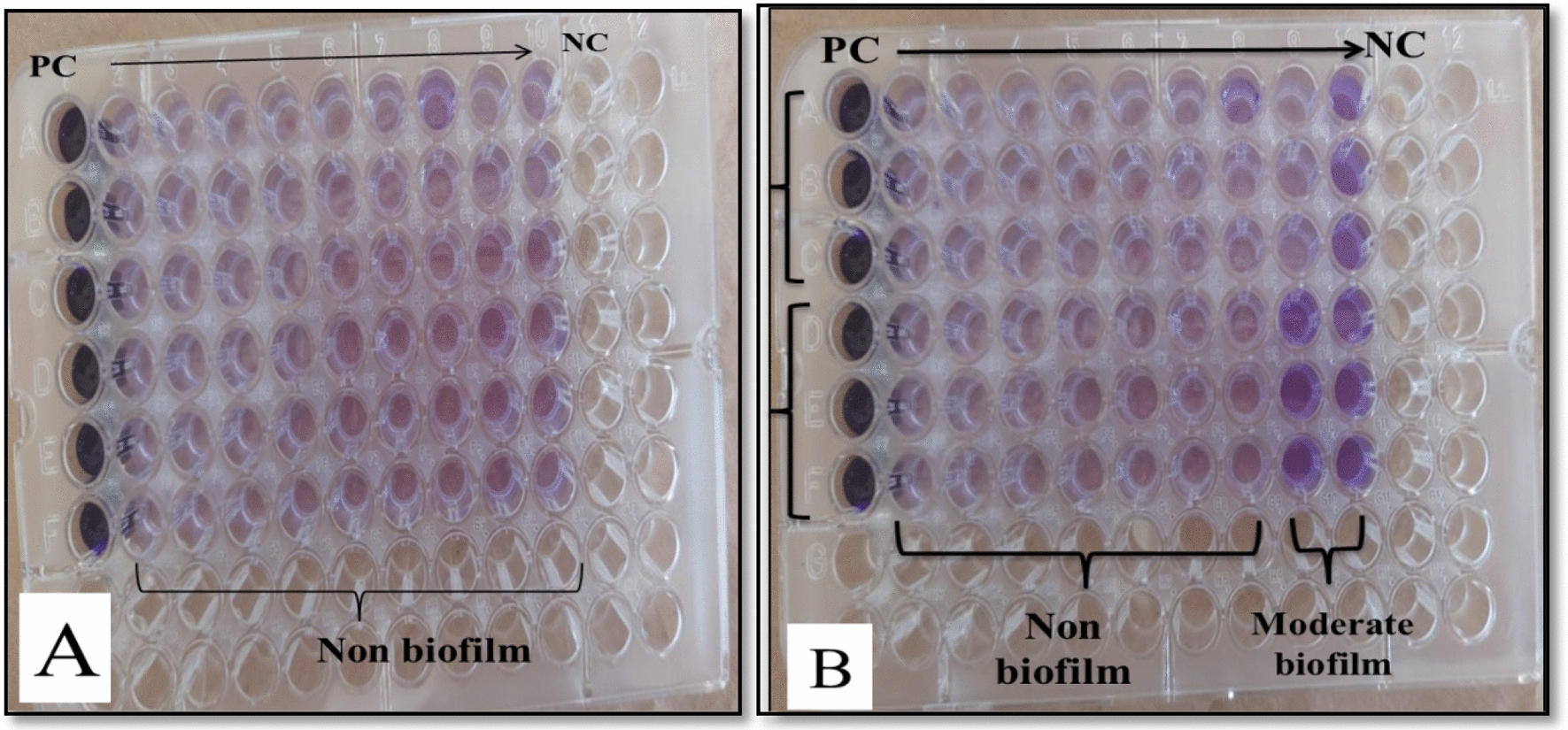
The biofilm inhibition effect of AgNPs by tissue culture plate method. **A** Antibiofilm effects of AgNPs on 2 strong biofilm producing strains of *S. aureus* tested in triplicate, the all tested concentrations of AgNPs changed the strong biofilm to non-biofilm. **B** Antibiofilm effects of AgNPs on 2 strong biofilm producing strains of *P. aeuriginosa* tested in triplicate. PC (positive control), concentrations of AgNPs from left to right (250, 125, 62.5, 31.25, 15.6, 7.8, 3.9, 1.9, 0.9 μg/mL), NC (negative control)

Variations in the biofilm formation profiles in *S. aureus* and *P. aeruginosa* both before and after AgNP treatment were evaluated. As regarding *S. aureus*, an obvious biofilm inhibition effect of AgNPs was observed at concentrations of 250, 125, 62.5, 31.2, 15.6, 7.8 and 3.9 μg/mL, a median of strong biofilm production before treatment with AgNPs significantly shifted to non-biofilm formation after treatment (*p* < *0.0001*) (Fig. 7A, Table 2). The median of biofilm categories is changed from strong to non-biofilm after treatment of *P. aeuroginosa* with the following concentration of AgNPs 250, 125, 62.5, 31.2, 15.6, 7.8 μg/mL (*p* < *0.0001*) (Fig. 7B, Table 2).

**Table 2:** Variations in the biofilm formation profiles in *S. aureus* and *P. aeuroginosa* before and after treatment with different concentrations of AgNPs

*S. aureus*				*P. aeuroginosa*			
Before treatment Median (range)	After treatment Median (range)	Wilcoxon signed rank test	*p* value	Before treatment Median (range)	After treatment Median (range)	Wilcoxon signed rank test	*p* value
At sliver concentration 250		Z = 5.28	<0.0001	At sliver concentration 250		Z = 6.03	<0.0001
3 (3:3)	0 (0:3)			3 (2:3)	0 (0:3)		
At sliver concentration 125		Z = 5.43	<0.0001	At sliver concentration 125		Z = 6.06	<0.0001
3 (3:3)	0 (0:2)			3 (2:3)	0 (0:3)		
At sliver concentration 62.5		Z = 5.61	<0.0001	At sliver concentration 62.5		Z = 6.07	<0.0001
3 (3:3)	0 (0:3)			3 (2:3)	0 (0:3)		
At sliver concentration 31.25		Z = 5.64	<0.0001	At sliver concentration 31.25		Z = 5.99	<0.0001
3 (3:3)	0 (0:2)			3 (2:3)	0 (0:3)		
At sliver concentration 15.6		Z = 5.64	<0.0001	At sliver concentration 15.6		Z = 5.85	<0.0001
3 (3:3)	0 (0:2)			3 (2:3)	0 (0:3)		
At sliver concentration 7.8		Z = 5.56	<0.0001	At sliver concentration 7.8		Z = 5.86	<0.0001
3 (3:3)	0 (0:1)			3 (2:3)	0 (0:3)		
At sliver concentration 3.9		Z = 5.56	<0.0001	At sliver concentration 3.9		Z = 5.74	<0.0001
3 (3:3)	0 (0:2)			3 (2:3)	1 (0:3)		
At sliver concentration 1.9		Z = 5.23	<0.0001	At sliver concentration 1.9		Z = 5.26	<0.0001
3 (3:3)	1 (0:3)			3 (2:3)	2 (0:3)		
At sliver concentration 0.9		Z = 4.75	<0.0001	At sliver concentration 0.9		Z = 3.16	0.002
3 (3:3)	2 (0:3)			3 (2:3)	3 (0:3)		

In contrast, eradication of biofilm formed by *S. aureus* was detected at 3.9 μg/mL, a low quantity of SeNPs*.*, with an inhibition value of around 80.2%. However, *P. aeruginosa* had the opposite results, the best biofilm inhibition capacity of SeNPs with a percentage of inhibition around 77.1% was attained with a higher SeNPs concentration of 31.25 μg/mL.

In the present study, gram positive and gram negative bacteria show the same variations in the biofilm formation profiles on exposure to SeNPs. For example, none of the concentrations of SeNPs changed the median of strong biofilm formation before treatment to non-biofilm category after treatment of either *S. aureus* or *P. aeruginosa* (Tables 3). Also, exposure of both *S. aureus* and *P. aeruginosa* to the following concentrations of SeNPs 15.6, 7.8 and 3.9 μg/mL, shifted the median from strong to weak biofilm.

**Table 3:** Variations in the biofilm formation profiles in *S. aureus* and *P. aeuroginosa* prior to and after treatment with different concentrations of SeNPs

*S. aureus*				*P. aeuroginosa*			
Before treatment Median (range)	After treatment Median (range)	Wilcoxon signed rank test	*p* value	Before treatment Median (range)	After treatment Median (range)	Wilcoxon signed rank test	*p* value
At selenium concentration 250		Z = 3.75	0.0002	At selenium concentration 250		Z = 6.03	<0.0001
3 (3:3)	3 (0:3)			3 (2:3)	2 (0:3)		
At selenium concentration 125		Z = 4.74	<0.0001	At selenium concentration 125		Z = 5.69	<0.0001
3 (3:3)	2 (0:3)			3 (2:3)	2 (0:3)		
At selenium concentration 62.5		Z = 5.05	<0.0001	At selenium concentration 62.5		Z = 5.41	<0.0001
3 (3:3)	2 (0:3)			3 (2:3)	2 (0:3)		
At selenium concentration 31.25		Z = 5.04	<0.0001	At selenium concentration 31.25		Z = 5.54	<0.0001
3 (3:3)	2 (0:3)			3 (2:3)	2 (0:3)		
At selenium concentration 15.6		Z = 5.13	<0.0001	At selenium concentration 15.6		Z = 5.15	<0.0001
3 (3:3)	1 (0:3)			3 (2:3)	1 (0:3)		
At selenium concentration 7.8		Z = 5.13	<0.0001	At selenium concentration 7.8		Z = 5.59	<0.0001
3 (3:3)	1 (0:3)			3 (2:3)	1 (0:3)		
At selenium concentration 3.9		Z = 5.22	<0.0001	At selenium concentration 3.9		Z = 5.64	<0.0001
3 (3:3)	1 (0:3)			3 (2:3)	1 (0:3)		
At selenium concentration 1.9		Z = 4.90	<0.0001	At selenium concentration 1.9		Z = 5.55	<0.0001
3 (3:3)	2 (0:3)			3 (2:3)	2 (0:3)		
At selenium concentration 0.9		Z = 3.71	0.0002	At selenium concentration 0.9		Z = 3.71	0.0002
3 (3:3)	3 (0:3)			3 (2:3)	3 (0:3)		

### AST pattern of *S. aureus* and evaluation of the effect of nanoparticles with antibiotics:

According to the results of AST, 100% of *S. aureus* isolates were resistant to cefoxitin (MRSA), The rate of resistance to various antibiotics was as follow; linzolid 94.44%, erythromycin 94.44%, clindamycin 94.44%, trimethoprim 94.44%, quinapristin 88.89%, rifampicin 88.89%, tetracyclines 83.33%, gentamycin 66.67%, ciprofloxacin 44.44%, chloramphenicol 44.44% and nitrofurantoin 33.33% (Table 4).

Combined effect of AgNPs with antibiotics on *S. aureus* was evaluated by disc diffusion method, we found that all antibiotics had synergistic antibacterial effect with AgNPs except gentamycin as shown in (Table 4), the mean of inhibition zone** ± **SD of gentamycin alone decreased from 8.75** ± **9.92 to 4** ± **3.85 after addition of AgNPs, thus gentamycin had antagonistic effect with AgNPs. Antibiotics which inhibit protein synthesis and affect metabolic pathways as erythromycin, trimethoprim, clindamycin and tetracycline had the strongest synergistic effect with AgNPs as represented by the increased fold area; 147%, 123.6%, 114.2% and 110.4% respectively (Table 4).

**Table 4:** Comparison of the inhibition zone of different antibiotics alone and inhibition zone of different antibiotics with silver and selenium nanoparticles on *S. aureus*

Modes of action	Percentage of antibiotic resistance (%)	Inhibition zone (mean ± SD) mm		Increased fold (%) with AgNPs	Inhibition zone (mean ± SD) mm	Increased fold (%) with SeNPs
		A	B		B	
		Antibiotic alone	Antibiotic + AgNPs	(B − A)/A) × 100	Antibiotic + SeNPs	(B − A)/A) × 100
Protein inhibition	Gentamycin 66.67%	8.75 ± 9.92	4 ± 3.85	− 50	18.72 ± 5.93	113.9
	Erythromycin 94.44%	7.22 ± 5.11	17.89 ± 6.19	147	13.11 ± 5.93	81.5
	Nitrofurantoin 33.33%	15.89 ± 7.86	20.39 ± 4.09	28.3	20.56 ± 5.50	29.3
	Tetracycline 83.33%	9.0 ± 6.81	19.28 ± 4.05	114.2	16.11 ± 6.25	79
	Clindamycin 94.44%	9.0 ± 6.21	18.94 ± 5.98	110.4	11.56 ± 7.28	28.4
	Chloramphenicol 44.44%	13.31 ± 11.30	22.44 ± 4.97	68.3	21.89 ± 5.30	64.4
	Quinapristin 88.89%	12.28 ± 6.79	20.11 ± 3.53	63.7	15.89 ± 6.48	29.2
	Linzolid 94.44%	13.11 ± 6.48	19.94 ± 4.20	52	15.39 ± 6.52	17.3
Metabolic path inhibition	Trimethoprim 94.44%	8.67 ± 6.97	19.39 ± 5.05	123.6	16.33 ± 7.75	88.3
Nucleic acid inhibition	Ciprofloxacin 44.44%	12.80 ± 9.78	20.22 ± 5.20	57.8	21.94 ± 5.58	71.4
	Rifampicin 88.89%	11.5 ± 6.27	20.17 ± 2.99	75.3	18.72 ± 4.56	62.7
Cell wall inhibition	Cefoxitin 100%	10.11 ± 4.65	16.44 ± 5.52	62.6	14.67 ± 4.43	45.1

Combined effect of SeNPs with antibiotics on *S. aureus* presented in (Table 4), all antibiotics had synergistic antibacterial effect with SeNPs. Gentamycin had the strongest synergistic effect, the increased fold area was 113.9%, followed by trimethoprim 88.3% erythromycin 81.5%, tetracycline 79%, and ciprofloxacin 71.4%.

### AST pattern of *P. aeuroginosa* and evaluation of the effect of nanoparticles with antibiotics

According to the results of AST on *P. aeuroginosa*, the resistance rate to antibiotics was as follow: aztreonam 91.30%, piperacillin 86.96%, ceftazidime 82.61%, ciprofloxacin 52.17%, piperacillin-tazobactam 47.83%, meropenem 47.83%, colistin 34.78%, impenem 21.74%, and gentamycin 21.74%.

All antibiotics had synergistic antibacterial effect with AgNPs on *P. aeuroginosa* (Table 5). The increased fold area for cell wall inhibiting antibiotics was as follow; 182% for piperacillin, 150% for ceftazidime, 120% for aztreonam, thus had the strongest synergistic effect with AgNPs. Impenem had antagonistic effect with SeNPs, the mean of inhibition zone** ± **SD decreased from 8.75** ± **9.92 for impenem alone to 4** ± **3.85 after addition of SeNPs to impenem, The other antibiotics had synergistic antibacterial effect with SeNPs. ceftazidime had the strongest synergistic effect, the increased fold area was 175%, followed by aztreonam 89.2% piperacillin 78%, and ciprofloxacin 77.8%.

**Table 5:** Comparison of the inhibition zone of different antibiotics alone and inhibition zone of different antibiotics with silver and selenium nanoparticles on *P. aeuroginosa*

Mode of action	Percentage of antibiotic resistance (%)	Inhibition zone (mean ± SD) mm		Increased fold (%) with AgNPs	Inhibition zone (mean ± SD) mm	Increased fold (%) with SeNPs
		A	B		B	
		Antibiotic alone	Antibiotic + AgNPs	(B − A) ⁄ A) × 100	Antibiotic + SeNPs	(B − A) ⁄ A) × 100
Cell wall inhibition	Piperacillin 86.96%	9.49 ± 6.79	17.35 ± 6.11	182	16.91 ± 5.22	78
	Pipertazobactam 47.83%	14.26 ± 8.75	20.70 ± 4.48	45	20.22 ± 5.10	41.7
	Ceftazidime 82.61%	5.61 ± 7.26	14.09 ± 8.03	150	15.48 ± 4.60	175
	Colistin 34.78%	11.17 ± 6.50	18.65 ± 3.52	66.9	19.65 ± 4.11	75.9
	Impenem 21.74%	19.26 ± 6.71	23.48 ± 5.39	21.9	16.30 ± 7.11	− 15.3
	Aztreonam 91.30%	7.70 ± 6.58	16.96 ± 6.87	120	14.57 ± 6.95	89.2
	Meropenem 47.83%	13.30 ± 11.24	21.78 ± 8.66	63.7	22.39 ± 7.03	68.3
Protein inhibition	Gentamycin 21.74%	11.5 ± 5.37	18.04 ± 4.49	48	20.13 ± 6.28	49
DNA inhibition	Ciprofloxacin 52.17%	12.57 ± 9.38	21.13 ± 6.21	67.8	22.35 ± 7.23	77.8

## Discussion

The pursuit of a practical application for microbial creating nanomaterials is an exciting new field of study for future sustainable industrial production. Fungi have also lately been touted as one of the potential bio-factories for producing a variety of nanoparticles [31]. that undoubtedly paves the doors for a number of industrial, agricultural, and medical applications [32, 33].

Therefore, the objective of the present study is the preparation of both AgNPs and SeNPs from *A. carneus* MAK 259, the colour change from colourless to brown and reddish orange indicate the formation of AgNPs and SeNPs, respectively. According to prior investigations on nanoparticles, the surface plasmon vibrations that these particles exhibit are what cause the alteration of nanoparticles' colour in aqueous solutions, this phenomenon occurs when conduction electrons on the nanoparticle surface resonate with incident light at specific wavelengths, leading to strong absorption and scattering, which causes the visible color change [34]. Several biomolecules, including enzymes, proteins, amino acids, exopolysaccharides, and vitamins, are incorporated in these extracts to reduce silver ions into silver nanoparticles. But the nitrate reductase enzyme found in the microbial extract is the most commonly accepted mechanism for the creation of AgNPs [35, 36]. A few papers have discussed the production of SeNPs by fungi [37, 38].

In the present study, the mixture of *A. carneus* MAK 259 extract with individual silver and selenite ions has a novel peaks associated with the successful synthesis of AgNPs at λmax = 410 nm which would correspond to spherical AgNPs [39] and SeNPs at λmax = 620 nm. Accordingly, SPR peaks between 400 and 450 nm have been reported for AgNPs produced by several microorganisms in previous works [12, 17]. Gangadoo et al. [40] approved the presence of stable selenium nanoparticles by UV–visible spectroscopy for SPR (262 nm). Hussein et al. [18] reported that the UV-spectrum of SeNPs formed by *A*. *quadrilineatus*, *A*. *ochraceus*, *A. terreus*, and *F*. *equiseti* had a peak in its maximum UV absorption at 265 nm.

The most precise technique for identifying the structural characteristics, such as the size and shape of the prepared AgNPs and SeNPs, is TEM analysis. The prepared nanoparticles has a spherical monodispersed shape with nanoscale size ranged from 5 to 26 nm in case of AgNPs and from 20 to 77 nm in case of SeNPs. Similarly, Xue et al. [24] and Gudikandula et al. [41] reported the ability of *Ganoderma enigmaticum* and *Trametes ljubarskyi* to create spherical AgNPs of 5–40 nm. According to these findings, the mycochemicals in the fungal filtrate act as stabilizers and capping agent, which regulated the size of the generated AgNPs [42]. Also, Zare et al. [38] reported that the size of the SeNPs was 47 nm with a spherical shape.

Using FTIR analysis, function groups at the surfaces of the generated AgNPs and SeNPs were investigated, and the results showed the chemistry of active function groups that may have a significant impact on the reduction of Ag and Se ions as well as their stability following reduction. Due to the binding of silver or selenium ions with the hydroxyl group, which is attributable to O–H and N–H stretching, the strong absorption was found at 3,424 cm^−1^ and indicates the presence of polyphenols. In addition, the presence of the “C=C” stretch at about 1633 cm^−1^ confirms that the synthesised nanoparticles contain a variety of alkene groups. The sharp bands at ~ 1404 and 1384 cm^−1^, were attributed to carboxylic functional groups. Another medium peaks at ~ 1037, 1045 cm^−1^ shows the existence of aliphatic amines due to C–N stretching. Our results matched those from other investigations [39, 43].

The crystallinity of AgNPs and SeNPs was confirmed using X-ray diffraction analysis that confirmed the occurrence of AgNPs and SeNPs. Additionally, Our data are consistent with the JCPDS card 257 no. 01-089-3722 published by Wu et al. [44], and the shape of the crystal is cubic with the space groups fm-3 m and 225. Our results in agreement with many previous studies [16, 19, 42, 43].

Additionally the physical and chemical stability of both AgNPs and SeNPs was assessed using zeta potential measurements that provide the possible information about the charges on the surface of nanomaterial, Herein the stability of nanoparticles was confirmed by the presence of negative potential of − 30 mV and − 25.7 mV, on the surface of both AgNPs and SeNPs, respectively. Also nanoparticles with negative zeta potential could influence the interaction between positive charged ions on microbial cell surfaces and their negative surfaces [19].

AgNPs have been demonstrated for its good antibacterial efficacy against many multidrug resistant microorganisms [15]. It was very clear that AgNPs were more efficient (*p* value 0.0006) against *P. aeuroginosa* (inhibition zone diameter mean ± SD = 29.83 ± 5.34) as compared to *S. aureus* (mean ± SD 26.28 ± 2.98). These results are consistent with Kokila et al. [45] and Saleh and Najim [46] who reported that AgNPs had a more antibacterial activity against Gram-negative bacteria than Gram-positive bacteria. This difference in the effectiveness of AgNPs in inhibiting the growth of *P. aeuroginosa* more than *S. aureus* were duo of variations in the cell wall structure of gram positive and gram negative bacteria. The cell wall of Gram-positive bacteria is a more rigid structure contains a thick peptidoglycan layer, which consists of linear polysaccharide chains bound by short peptides, resulting in difficult permeation of the NPs, while Gram-negative bacterial cell wall possesses a thinner layer of peptidoglycan [46]. The results of this study are in contrast with findings reported with Salman [47] and Rahimi et al. [48] that AgNPs had a more effective antibacterial activity against Gram-positive bacteria than Gram-negative bacteria.

Also, SeNPs were more efficient (*p* value < 0.0001) against *P. aeuroginosa* (inhibition zone diameter mean ± SD = 37.52 ± 3.54) as compared to *S. aureus* (mean ± SD 32.94 ± 3.01). These findings confirmed by findings published by Geoffrion et al. [49] who found that growth of *P. aeruginosa* inhibited by lower SeNPs concentrations around 100 ppm, while higher doses of SeNPs 125 ppm inhibit the growth of *S. aureus*.

It is evident from the antibacterial activity data of AgNPs and SeNPs that SeNPs were a more potent growth inhibitor for *P. aeuroginosa* and *S. aureus* (*p* value <0.0001, <0.0001) than AgNPs. The powerful antibacterial effect of SeNPs may be attributed to the mechanism of action of SeNPs in causing cell membrane disruption. At neutral pH, the cell wall gives both Gram-positive and negative bacteria a negative charge. However, Gram-negative bacteria have the highest negative charge. The interactions between the bacterial cell wall and the NPs and ions released from it are expected to be influenced by this negative charge [50]. The binding of SeNPs to the cell wall and membrane, which is dependent on the electrostatic attraction between the positively or less negatively charged SeNPs and the negatively charged microbial cell membrane, is the first step in the interaction between SeNPs and bacteria [51] leading to structural and morphological changes and membrane depolarization, followed by disruption of membrane permeability and respiratory functions, damage to the integrity of the cell, and ultimately, cell death [52].

AgNPs had been evaluated for anti-biofilm effect on biofilm producing bacteria In this study, AgNPs had a very highly significant antibiofilm effect (*p* value < 0.0001) against *S. aureus* at all concentrations between 250 and 0.9 μg/mL, the maximum biofilm inhibition value 94.36% was achieved at AgNPs concentration 15.6 μg/mL. These findings were agreed with the outcomes of Goswami et al. [53] who stated that biofilm eradication by AgNPs was achieved at concentration 15 μg/mL in *S. aureus* and produced an 89% inhibition of the formation of biofilms While, El-Shennawy et al. [16] revealed that a higher concentration of AgNPs at 100 μg/mL had the highest antibiofilm effect (82%).

Inconsistent to our results, Choi et al. [54] reported that bacterial biofilms are less vulnerable to AgNPs than planktonic cells, which might be explained by the presence of extracellular matrix. coated cells in the biofilm and the aggregated cells with reduced surfaces exposed to AgNPs.

As regarding MDR *P. aeruginosa*, higher concentration of AgNPs 62.5 μg/mL had the best biofilm inhibition capacity with inhibition value around 90.3%. Results of this study confirm that AgNPs were more efficient against biofilms produced by the *S. aureus* at reasonably low concentrations than MDR *P. aeruginosa*. Palanisamy et al. [55] reported that AgNP concentrations must be greater for MDR strains to prevent the production of biofilms.

In this study, SeNPs had a weaker antibiofilm effect than AgNPs, none of the concentrations of SeNPs changed the strong biofilm producters to non-biofilm producers of either *S. aureus* or *P. aeruginosa*. Otherwise, the antibiofilm effect of SeNPs on *S. aureus* and *P. aeruginosa* had the same pattern of AgNPs, eradication of biofilm formed by *S. aureus* was noted at a low SeNPs concentration of 3.9 μg/mL, with the greatest inhibition value of around 80.2%. However, *P. aeruginosa* had the opposite results, the best biofilm inhibition capacity of SeNPs with a percentage of inhibition around 77.1% was accomplished with a higher concentration of 31.25 μg/mL. In contrast, Ullah [56] showed that SeNPs had the greatest ability to disperse biofilms at a concentration of 700 µg/mL within 15 min against *P. aeruginosa* (85.7%) and *S. aureus* (78.3%). The discrepancy of these results may be attributed to difference in the nature, structure and source of SeNPs used in the study.

### Synergistic potential of antibacterial activity

In this study, All antibiotic combinations with AgNPs demonstrated significant (*p* < 0.0001) synergistic effects against *S. aureus*. except gentamycin, the increased fold area ranged from 52 to 147%. The combination of erythromycin with AgNPs demonstrated the greatest synergistic impact When compared to other antimicrobials.

The antibacterial effectiveness of each of the following antibiotics; tetracycline, trimethoprim-sulfamethoxazole, rifampicin and quinapristin increased synergistically in combination with AgNPs from resistant to susceptible range according to CLSI.

AgNPs exhibited moderate synergistic effect in combination with ciprofloxacin on *S. aureus* (increased fold 57.8%), Shokoofeh et al. [57] showed significant synergistic effects of Fe3O4@Ag nanocomposite prepared by *Spirulina platensis* Cyanobacterium and ciprofloxacin in MIC reduction with good impacts on the expression of efflux pump genes "norA and norB" in ciprofloxacin-resistant S. aureus (CRSA) more than twofold compared to control.

Kahzad and Salehzadeh [58] synthesized CuFe2O4@Ag nanocomposite using aqueous extract from microalgae *Chlorella vulgaris* which had synergistic antibacterial activity with ciprofloxacin against *S. aureus*. Also, the expression multidrug resistance efflux pumps, gene NorA among clinical and standard strains treated with CuFe2O4@Ag nanocomposite combined with ciprofloxacin reduced by 59% and 65%, respectively.

All antibiotics had synergistic antibacterial effect with AgNPs on *P. aeuroginosa*, the increased fold area ranged from 21.9 to 182%. The findings of this study unequivocally show that when an antibiotic is combined with AgNPs, antibiotic-resistant bacteria revert to susceptible state, as evidenced by the synergistic effect of merpenem and gentamycin with AgNPs against *P. aeuroginosa*. Similar to these findings, according to the results of the research by Panácek et al. [59] on *P. aeuroginosa*, the synergetic effect of AgNPs was proved with merpenem,,gentamycin, and colistin.

The mechanisms resulting in increase of bacterial sensitivity to antibiotics used in combination with AgNPs and restoration of sensitivity of bacteria initially resistant to antibiotics may be of multiple nature keeping in mind the AgNPs' numerous modes of action. For example, AgNPs and antibiotics can collaborate together to cause bacterial cell wall disruption or direct cell wall damage. AgNPs may improve the membrane's permeability, making it easier for antibiotics to enter bacterial cells. Also, AgNPs have inhibitory effect on enzymes produced by antibiotic resistant bacteria, such as carbapenemases and B-lactamases which can bind to the surface of NPs resulting in modification of their structure and affection of their functions. Additionally, ionic silver released from AgNPs may inhibit enzymatic activity [59].

The use of AgNPs in conjunction with antibiotics may increase the drug's efficacy by increasing the affinity of drug binding to the target structure and improve drug penetration through the cell wall this occur because AgNPs may transport more medications due of their tiny size and broad surface area, raising the concentration of antibiotics at the site of contact between antibiotic and bacteria, and can further suppress bacteria [60, 61].

In this research, we observed an important and interesting finding, the antibiotic classes which gave synergistic effects in combination with AgNPs on Gram-positive and Gram-negative bacteria were different. On *P. aeuroginosa*, AgNPs had higher synergism in combination with cell wall inhibiting antibiotics as piperacillin, ceftazidime and aztreonam.

DNA inhibitor antibiotics "ciprofloxacin" show moderate Synergistic effect on *P*. *aeuroginosa* in combination with AgNPs (increased fold 67.8%), while Abdolhosseini et al. [62] found that silver nanoparticles functionalized by Thiosemicarbazid (MIC ≥ 32 μg/mL) displayed synergistic effects with ciprofloxacin which efficiently inhibited bacterial growth with simultaneous reduction in the expression of ciprofloxacin resistance genes "mexA and mexB" by 6.0 and 2.75 folds, respectively.

While, higher synergestic effects of AgNPs against *S. aureus* were observed in combination with antibiotics that inhibit protein synthesis and affect metabolic pathways as erythromycin, trimethoprim-sulfamethoxazole, clindamycin and tetracycline. Consequently, the synergistic efficiency of AgNPs with antibiotics is influenced by the difference in the composition of the bacterial cell wall between Gram- positive and Gram- negative bacteria.

In contrast to our findings, Panácek et al. [59] reported that no specific patterns were noticed for the synergistic impacts of antibiotics classes with variable mechanisms of action combined with AgNPs against Gram positive and Gram negative organisms, demonstrating the non-specific synergistic impacts of AgNPs + antibiotics. Krishnaraj et al. [63] reported that AgNPs do not impact bacteria through a single mechanism of action, like breaking down the bacterial cell wall, inhibiting protein synthesis or preventing synthesis of nucleic acid which should result in enhanced synergistic impacts with antibiotics have certain mechanisms of action. Furthermore, AgNPs change membrane permeability, impact purine's metabolism and destroy the important metabolic pathways of bacteria [63].

All antibiotics had synergistic antibacterial effect with SeNPs. on *S. aureus*. Higher synergestic effects were observed in combination with antibiotics that inhibit protein synthesis and affect metabolic pathways as gentamycin, trimethoprim, erythromycin, tetracycline. All antibiotics had synergistic antibacterial effect with SeNPs on *P. aeuroginosa* except impenem which had antagonistic effect with SeNPs. As well as AgNPs, the SeNPs gave higher synergestic effects in combination with cell wall inhibiting antibiotics as ceftazidime, aztreonam and piperacillin.

## Conclusion

The current work exemplifies a quick and practical approach for synthesis of Both AgNPs and SeNPs using *Aspergillus carneus* MAK 259. AgNPs as well as SeNPs had more effective antibacterial effect against *P. aeruginosa* than *S. aureus*. SeNPs had a more remarkable inhibitory effect on the growth of both organisms than AgNP_S_. On *S. aureus*, obvious biofilm inhibition effect of AgNPs was observed at concentrations from 250 to 3.9 μg/mL*.* On *P. aeuroginosa*, biofilm eradication was noticed with AgNPs concentrations from 250 to 7.8 μg/mL*.* None of the concentrations of SeNPs changed the median of strong biofilm production to non-biofilm category after treatment of either *S. aureus* or *P. aeruginosa.* Both NPs had higher synergestic effects against *P. aeuroginosa* in combination with cell wall inhibiting antibiotics and on *S. aureus* in combination with antibiotics that inhibit protein synthesis and metabolic pathways.

The described results would likely spur further research into understanding the precise mechanisms of action of these nanoparticles and their interactions with bacterial cells. Additionally, efforts may be directed towards optimizing synthesis processes, exploring additional medical applications, and conducting clinical trials to assess the efficacy of these nanoparticles in humans.

## Supplementary Information

Additional file1

## Data Availability

The raw sequencing data analysed during the current study are available in the National Center for Biotechnology Information (NCBI) repository under the accession number OR480101. All other data generated or analysed during this study are included in this manuscript [and its supplementary information files].
